# Applications of Inorganic Nanoparticles in Food Packaging: A Comprehensive Review

**DOI:** 10.3390/polym14030521

**Published:** 2022-01-27

**Authors:** Kshirod Kumar Dash, Pinky Deka, Sneh Punia Bangar, Vandana Chaudhary, Monica Trif, Alexandru Rusu

**Affiliations:** 1 Department of Food Processing Technology, Ghani Khan Choudhury Institute of Engineering and Technology Malda, Kolkata 732141, India; 2 Department of Applied Biology, University of Science & Technology, Techno City 793200, India; pinkydeka93@gmail.com; 3 Department of Food, Nutrition and Packaging Sciences, Clemson University, Clemson, SC 29634, USA; snehpunia69@gmail.com; 4 Department of Dairy Technology, Lala Lajpat Rai University of Veterinary and Animal Sciences, Hisar 125001, India; dhakavandana18@gmail.com; 5 Centre for Innovative Process Engineering (CENTIV) GmbH, 28857 Syke, Germany; monica_trif@hotmail.com; 6 Life Science Institute, University of Agricultural Sciences and Veterinary Medicine Cluj-Napoca, 400372 Cluj-Napoca, Romania

**Keywords:** nanocomposite, food packaging, metal oxides, gold nanoparticles, copper nanoparticles, zinc nanoparticles

## Abstract

Nanoparticles (NPs) have acquired significance in technological breakthroughs due to their unique properties, such as size, shape, chemical composition, physiochemical stability, crystal structure, and larger surface area. There is a huge demand for packaging materials that can keep food fresher for extended periods of time. The incorporation of nanoscale fillers in the polymer matrix would assists in the alleviation of packaging material challenges while also improving functional qualities. Increased barrier properties, thermal properties like melting point and glass transition temperatures, and changed functionalities like surface wettability and hydrophobicity are all features of these polymers containing nanocomposites. Inorganic nanoparticles also have the potential to reduce the growth of bacteria within the packaging. By incorporating nano-sized components into biopolymer-based packaging materials, waste material generated during the packaging process may be reduced. The different inorganic nanoparticles such as titanium oxide, zinc oxide, copper oxide, silver, and gold are the most preferred inorganic nanoparticles used in food packaging. Food systems can benefit from using these packaging materials and improve physicochemical and functional properties. The compatibility of inorganic nanoparticles and their various forms with different polymers make them excellent components for package fortification. This review article describes the various aspects of developing and applying inorganic nanoparticles in food packaging. This study provides diverse uses of metals and metal oxides nanoparticles in food packaging films for the development of improved packaging films that can extend the shelf life of food products. These packaging solutions containing nanoparticles would effectively preserve, protect, and maintain the quality of the food material.

## 1. Introduction

Food packaging serves to preserve, protect, and maintain quality of food products, subsequently reducing food waste. Most of the currently employed packaging materials are nondegradable and often lead to environmental issues [1]. This problem could be resolved by using edible and biodegradable films, which will decrease the waste production caused by packaging material and increase the shelf life and the quality of food commodities [2]. However, a very restricted group of bio-based materials are employed to label food products. These restrictions in the packaging are attributed to the low barrier and mechanical properties. Additionally, in many bio-based food packaging systems, permeability and moisture migration are major concerns. Decreased oxygen permeability reduced oxygen levels in the package headspace that accelerated deterioration in packaged shrimp [3,4]. Improving oxygen barrier properties would increase the utilization of biodegradable packaging to preserve the quality of packaged products [5]. Atmospheric moisture cannot be restricted completely by any packaging material. In contrast, in some other food materials, a complete barrier for the permeation of gases is not required, such as in fresh fruits and vegetables, as these food items undergo cellular respiration. In the case of beverages packaging, a high barrier to gases like oxygen and carbon dioxide is required to limit oxidation and de-carbonation of the beverage. Nanotechnology has led to an overall revolution in packaging different processed and fresh commodities. The formulation of nanocomposites with nanoscale fillers in a biopolymer matrix might assist to minimize many issues with their application and functional qualities.

Nanotechnology encompasses manipulation, fabrication, and characterization of the nano-scale sizes and structures, ranging from 1 to100 nm in length. The particle size reduction to nanoscale leads to a broad deviation of the physical and chemical properties from that of the properties displayed by the particles with sizes at the macro or micro scale [6]. It also contributes to the moderation of the overall nutritional confirmation of the final products. Several companies throughout the globe are using nanotechnology in the manufacturing of food packaging systems [7,8]. Nanotechnology can play an important role in detecting the growth or presence of bacteria within food packaging. Using nanotechnology, the production of stronger flavor can also be moderated, thus, leading to the maintenance of the quality of the product through sensing its barrier properties [9]. Using different nanocomposites, the functions of edible and biodegradable films can be improved [10,11]. In this process, nanoparticles incorporated in the films increase the shelf life of the foods by regulating the exchange of gases across the films. This also aids in the elimination of undesirable gases from packaged goods, the presence of which may reduce the shelf life of the food product [12].

Inorganic nanoparticles have the potential to be incorporated with film forming solution to produce nanocomposite films with better characteristics as food packaging. The ability of nanomaterials is influenced by elements such as nanoparticle form, size, concentration, surface charge, metal ion release, and the type of surrounding media. Therefore, different scenarios must be considered for the formulation and application of packaging materials. Based on this, the review focuses on the applications of various metal and metal oxide nanoparticles and the variables influencing their effectiveness in various packaging applications to preserve and increase functional and physiochemical attributes in food systems. This review provides an overview of the nanotechnologies applied to design innovative food packaging, with a particular emphasis on enhancement of physical, mechanical, and antimicrobial properties of the film.

## 2. Application of Nanoparticles

The following sections discuss the applications of different metal nanoparticles such as silver nanoparticles, gold nanoparticles, and metal oxide nanoparticles (zinc oxide, titanium dioxide, copper oxide, and silicon dioxide) in various packaging materials.

### 2.1. Silver Nanoparticles

**Figure 1:**
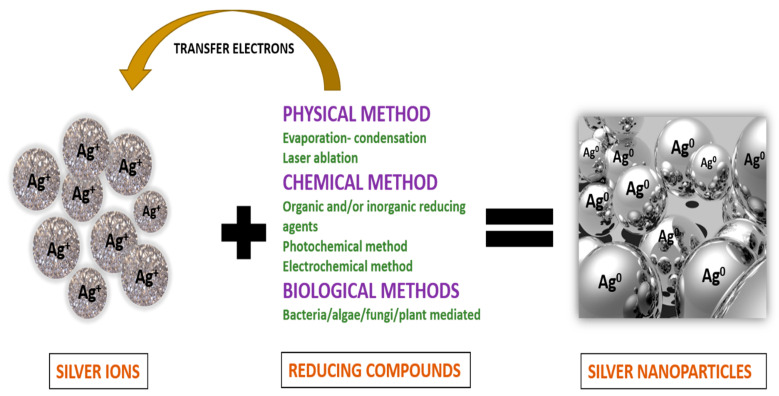
Methods for preparation and stabilization of silver nanoparticles.

Silver nanoparticles (AgNPs) are more efficient against pathogenic microbes such as viruses, bacteria, yeast, and fungus [13,14,15]. The antagonistic action of silver nanoparticles (AgNPs) against microorganisms can be attributed to two forms, which are the Ag^0^ and Ag^+^ species. Several physical, chemical, or biological methods, as depicted in Figure 1, are employed to orchestrate and stabilize silver nanoparticles [16].

In many studies, it has been found that biopolymers act as potential media for the development and stabilization of AgNPs [17,18,19]. The retention of a specific nanostructure attribute such as aggregation, composition, crystallinity, shape, size, and surface chemistry is referred to as nanoparticle stability. The adsorption of a dispersion layer around the particle surface is often used to stabilize nanoparticles. The creation of an adequate thickness dispersion layer is critical for the stability of solutions with high nanoparticle concentrations. The nanofilms can be synthesized in two ways: in situ and ex situ. In the case of the in situ method, for the formation of AgNPs, the polymer acts as a medium for the reaction and stabilization as well. In the case of the ex situ method, the matrix of polymer acts as a medium for dispersion and stabilization for the AgNPs developed earlier [20]. In one of the studies, it was observed that dextran was used as a medium for developing edible films and coating for the in situ development of AgNP with a size of 10–60 nm. In doing so, the AgNPs got capped properly with the dextran molecules by joining together with the -OH group of dextran, thus preventing its aggregation and providing stability for a month or more. Therefore, the corresponding AgNPs showed good antibacterial activity against many microorganisms like *S. aureus*, *B. cereus*, *P. aeruginosa*, *B. subtilis*, and *E. coli* [21].

Another study observed that when a chitosan film with AgNPs was produced in situ, it demonstrated well-dispersed properties and stability for almost three months [22,23]. The metal formed a strong bond with the amine and the hydroxyl groups of chosen substrate [24]. Therefore, it showed strong antimicrobial activity against both Gram-positive and Gram-negative bacteria.

The AgNPs can also be added into the polymeric matrix by the ex situ method to achieve good antimicrobial properties. For example, when a study was carried out with AgNPs incorporated into hydroxypropyl methylcellulose polymer, it displayed good antimicrobial properties against different bacteria [25]. It was also found that when a composite film consisting of HPMC (hydroxypropyl methylcellulose), tragacanth, and beeswax was prepared with incorporation of AgNPs, a successful inhibition against pathogens including *E. coli*, *B. cereus*, *S. aureus*, *S. typhimurium*, *K. pneumonia*, and *P. aeruginosa* was observed [26]. The antimicrobial effect of Ag nanoparticle-based nanofilms depends upon many factors such as the size of particles, shape, concentration in the film, and its interaction with the polymer matrix. In a study by Moura et al. (2012), an antimicrobial film made up of hydroxypropyl methylcellulose with AgNPs was observed to be more effective against *E. coli* and *S. aureus* when the size of AgNPs was 41 nm as compared to 100 nm. This effect was due to the increased surface area, which ultimately increased interaction with the microorganisms [25]. Silver nanoparticles can attach to the bacterial cell wall and subsequently penetrate through it, causing structural changes in the cell membrane, such as membrane permeability and cell death. In a study, it was found that when a higher percentage of AgNPs was incorporated in the pullulan film and tested against *Aspergillus niger*, 76% inhibition occurred when AgNPs was 1.710 mg/g, followed by 45%, 22%, and 12% inhibition observed at the AgNPs concentrations of 0.803 mg/g, 0.317 mg/g and 0.156 mg/g of film, respectively [27]. Additionally, when 100 nm AgNPs were incorporated in pullulan at a concentration of 2%, it formed inhibition zones of 25–30 mm against *L. monocytogenes* ATCC 94,229 and 15–23 mm against *S. aureus* ATCC 11,988 [28]. In the case of guar gum incorporated with <100 nm size AgNPs, a reduction of *L. monocytogenes ATCC* 19,114 count was found to be 0.5 log CFU/mL at a concentration of 7.5 mg of AgNP, 2.0 log CFU/mL at a concentration of 15 mg, and 3.5 log CFU/mL at a concentration of 30 mg of AgNPs. Therefore, it can be concluded that with the increase in the concentration of AgNPs in the film, the antimicrobial activity increases. These activities of AgNPs can be seen due to the release of AgNPs from the film and the corresponding reactions with the microorganisms. The interaction of Ag nanoparticles with the peptidoglycan cell wall and plasma resulted in antibacterial activity. The Ag nanoparticles impair the bacterial membrane permeability by triggering the formation of many pits and holes, indicating that the bacterial cell membrane structure has been disrupted. The mechanism generally consists of steps like building up of AgNPs on the cell surface followed by destabilization of the cell membrane of bacteria due to the development of gaps. Through these gaps, cytoplasmic leakage occurs, and thus AgNPs get attached to the biomolecules, including protein and nucleic acid. It leads to the destruction of the functionality of the developed reactive oxygen species [29]. The bactericidal activity of a film formed of pullulan combined with AgNPs was also reported to be sustained when stored at a temperature less than 25 °C, indicating that temperature is also a significant factor regulating the growth of bacteria in film [28].

Polysaccharides and protein are mostly used to prepare biopolymer-based packaging materials. It has been observed that biopolymers are transparent, but their transparency is diminished when they are combined into AgNPs. In a study using green methodologies, a carrageenan film with AgNPs was prepared with yellowish-brown color, attributed to the film by AgNPs [30]. Furthermore, the addition of AgNPs improved the film’s barrier properties against UV light and improved thermal stability with the change in color.

Some studies found that the water vapor transmission value of some biopolymers decrease when AgNPs were incorporated in the films [25,26]. However, in a study carried out by Roy et al. (2019), it was found that with the increase in the concentration of AgNPs, the water vapor permeability also increased for carrageenan films. A similar result was also obtained for agar film [31]. Some studies on the applications of films and coatings incorporated with AgNPs are discussed in Table 1.

### 2.2. Gold Nanoparticles

Gold nanoparticles are also called gold colloids, wherein the size ranging between 1 and 100 nm shows greater stability. Due to their unique properties, they offer widespread applications. They have well-defined optical properties due to the collective oscillation of electrons on their surface. This property can be modified or précised by controlling its size, composition, and chemistry. As these nanoparticles have greater compatibility with various active molecules, their immobilization causes a minor effect on their functional activities. Because of their huge surface area, gold nanoparticles (AuNPs) serve as an excellent scaffold for the immobilization of various amounts of functional groups, resulting in increased sensitivity of targeted molecules [49]. They are inert and have high resistance to surface oxidation [50]. AuNPs possess potent antimicrobial activity depending on the sizes and shapes of the nanoparticles [51]. AuNPs incorporated in various polymer materials play a vital role in the active packaging of food [52,53]. Many investigations have established that bacteria with foodborne, respiratory chain enzyme activity experience structural changes that cause harm and, eventually, cell death [54]. It has been observed that the attachment of these gold nanoparticles to the DNA of bacteria retards the uncoiling of DNA, leading to the prevention of DNA transcription [55,56]. The combination of the gold nanoparticles with antibiotics could improve the antibacterial activity. This observation has been supported by a study by William et al. (2006), wherein when the gold nanoparticles were covered with vancomycin, corresponding antibacterial activity was improved. Quinoa starch-based films were prepared by imbibing gold nanoparticles; it was observed that films could inhibit the proliferation of *E. coli* and *S. aureus* by 99 and 98 percent, respectively [52]. Different materials with gold nanoparticles and their effects on various microorganisms are summarized in Table 2.

## 3. Metal Oxide Nanoparticles for Food Packaging Application

**Figure 2:**
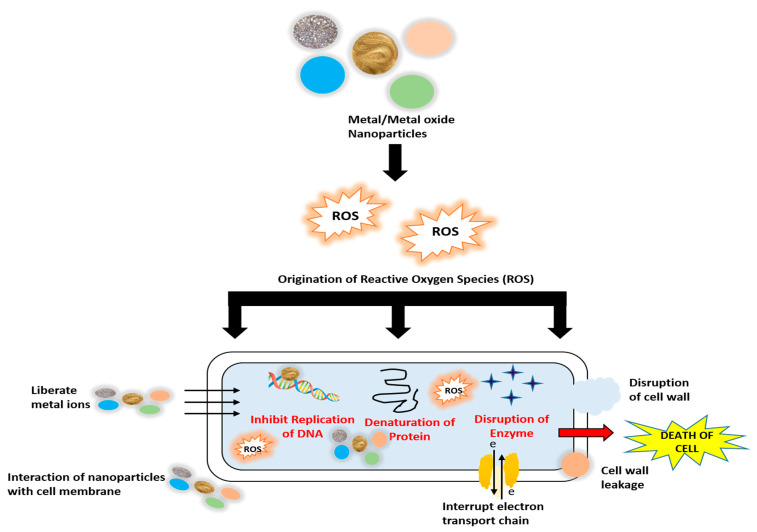
Mechanism of action of metal and metal oxide nanoparticles on bacterial cells.

Metal oxide nanoparticles have a higher surface area due to their smaller size, which makes them useful in various applications such as biosensors, bio-nanotechnology, and nanomedicine [61]. These nanoparticles have many atoms on their surfaces, making them highly reactive. Different features of these nanoparticles are attributed to their crystallinity, size, content, and shape. They can easily enter the inner structures of cells and interact with cell biomolecules [62]. The mechanism of action of nanoparticles on bacterial cells is shown in Figure 2 [63].

### 3.1. Zinc Oxide (ZnO) Nanoparticles for Food Packaging

ZnO has a wide range of applications because of its strong optical, electrical, piezoelectrical, semiconducting, and chemical sensing capabilities [64,65]. It was observed that increased antibiotic resistance of bacteria, its mutation, and unavailability of vaccine causes many health hazards to human beings. The high annual mortality rate has been attributed to the consumption of food with bacterial contamination. Hence, the development of antibacterial agents against food pathogens such as *Salmonella typhii*, *Clostridium perfringes*, and *Pseudomonas aeruginosa*, etc. have become an important objective for current research. Studies have been carried out to examine the antibacterial activity of ZnO nanoparticles and the viability of the bacteria under ZnO applied conditions. Also, factors related to their antibacterial activity, mechanism of toxicity of ZnO nanoparticles towards these bacteria, and their applications in food have been studied during several research projects. It has been found in many studies that ZnO acts as an antibacterial agent when it is reduced to its micrometer as well as nanometer size. ZnO nanoparticles react with the surface and core of the bacteria, thus causing a bactericidal effect [66]. These nanoparticles also show high photochemical activities and catalytical activities along with antibacterial and antifungal properties. As ZnO absorbs UVA in the range of 315–400 nm and UVB in 280–315 nm, it enhances the antibacterial responses [67].

The antibacterial property of ZnO nanoparticles is also influenced by their concentration and particle size. In many studies, it has been seen that the antibacterial activity increased with an increase in surface area and concentration [68,69]. With a decrease in the size, the specific surface area gets increased. Hence, ZnO nanoparticles can easily penetrate the bacterial cell membranes [70,71,72]. It was also found that with an increase in the concentration of ZnO nanoparticles, the death of cells increased. When the concentration increased, it caused interruption of the function of mitochondria, leakage of lactate dehydrogenase, and a change in the morphology of the cell [73]. Thus, it can be concluded that increased surface area and concentration lead to increased antibacterial activity of ZnO nanoparticles. In one study, it was shown that when the concentration of ZnO particles was kept 2 mM along with small-sized nanoparticles, the growth of bacteria was reduced by 99% [74]. In another study on oral bacteria, ZnO demonstrated bacteriostatic effect against *Lactobacillus salivarius* and *Streptococcus sobrinus*, and showed inhibitions against other strains, i.e., *P. aeruginosa*, *Streptococcus mutans*, and *S. aereus* [73]. E. coli growth was inhibited when it was exposed to a concentration of 10 mM ZnO for 30 min [75]. When *Salmonella enterica*, *Enter idis*, other *Salmonella* strains, and *E. coli O157:H7* were exposed to a lower concentration of ZnO nanoparticles with size 30 nm, a total of 100% bactericidal effect was observed [76].

One of the mechanisms on which the antibacterial activity of ZnO nanoparticles depends is reactive oxygen species formation. In one of the studies, it was observed that exposure of ZnO nanoparticles to UV produces reactive species such as hydrogen peroxide, hydroxide, and superoxide anions, which cause damage to cellular components, i.e., proteins, lipids, and DNA, and cause internalization of cell membrane of bacteria [77]. The antibacterial activity of ZnO nanoparticles is also based on the mechanisms of release of Zn ions in the medium consisting of both ZnO nanoparticles and bacteria [78,79,80], wherein the released Zn ions (from the ZnO nanoparticles) are responsible for its toxicity. This toxic interaction leads to their widespread application, mostly in the food packaging industries. It has also been observed that ZnO nanoparticles have toxic interaction with bacteria but are not toxic to human cells [81].

The morphology of ZnO nanoparticles affects the internalization mechanism. Different morphologies of ZnO nanoparticles are produced depending on the application; hence, the synthesis technique varies. These ZnO nanoparticles are manufactured using several chemical and/or physical procedures, but the chemical method is favored as it provides precise control of the shape and size of the nanoparticles [82]. Methods of fabrication require the use of various parameters that may be chemical or physical, including type of solvent, pH, temperature, etc. [83]. ZnO nanoparticles are available in different configurations compared to other metal oxides, including nano-cages, nano-combs, nano-helixes, nano-belts, etc. [84]. In addition, by modifying the growing conditions, different shapes of ZnO nanoparticles can be obtained, such as flowers, spheres, snowflakes, boxes, plates, spirals, drums, etc. [85]. The wire and rod-shaped ZnO nanostructures can penetrate the bacterial cells more easily than the spherical shaped ZnO nanoparticles [72]. It has also been shown that the biocidal activity of flower-shaped ZnO nanoparticles is higher than that of spherical and rod-shaped ZnO nanoparticles [86]. Different methods for achieving various ZnO nano-particles are summarized in Table 3.

A study was carried out to develop packaging films made up of agar incorporated with zinc nanoparticles to improve mechanical and functional properties. Being transparent and flexible, agar can be used to synthesize packaging films. ZnO nanoparticles were made from the extract of plant *Mimusops elengi* and added to the agar matrix. The effectiveness of the package was evaluated by observing the external features of packaged green apples in the agar-nanoparticle-based packaging material under ambient conditions. Two packaging films were prepared with 2% ZnO nanoparticles and 4% ZnO nanoparticles. Green grapes wrapped in plastic (polyethylene) film spoiled after 7 days due to mold development and leaking of sticky liquid, but fruits wrapped in agar-ZnO films with 2% ZnO nanoparticles remained fresh even after 14 days and for 21 days when wrapped in films with 4% ZnO nanoparticles [92]. As a result, a combination of ZnO nanoparticles and suitable coatings might be beneficial in food packaging. In another study, ZnO NPs incorporated in gelatin-based composite films demonstrated significant antibacterial activity against Gram-positive and Gram-negative food pathogens. Moreover, permeance to water and elongation at break of ZnO NPs incorporated films increased, whereas tensile strength and modulus of elasticity decreased [31].

### 3.2. Titanium Dioxide (TiO_2_) Nanoparticles for Food Packaging

Titanium dioxide (TiO_2_) shows antimicrobial activity against several food-borne pathogens, including *Vibrio parahaemolyticus*, *Listeria monocytogenes*, and *Salmonella enterica* even under the UV light [93]. TiO_2_ has great potential for controlling food hazards in food industries. Under UV irradiation, TiO_2_ acts as a scavenger of oxygen; therefore, it can be used to control food spoilage caused by oxygen [94]. It produces reactive oxygen species after absorbing photo energy, which is useful to kill microbes [95]. It can also absorb wavelength light, thus acting as a good UV blocking material. In addition to photostability, it also helps keep transparency in the food packages. Due to its photocatalytic activity under UVA or irradiation of black light, it has the power of self-cleaning that leads to antibacterial effects [92].

Agglomeration of TiO_2_ nanoparticles affects the functional property of films. This property of TiO_2_ nanoparticles is modified by improving its surface properties. During a study, the solvent evaporation method was used to prepare a biodegradable film using fish skin gelatin and TiO_2_ nanoparticles to achieve the required surface properties [96]. Different methods have also been employed during various studies focused on the application of TiO_2_ nanoparticles. TiO_2_ nanotubes were synthesized using a deposition process, where atomic layers covered electrospun PVA (polyvinyl alcohol) nanofibers at different temperature levels to obtain nanostructures with antibacterial properties and large selective area [97]. Another study used the sol-gel method to synthesize eco-friendly pectin –TiO_2_ nanocomposite aerogels, wherein initially pectin was dissolved in water followed by the addition of a measured amount of TiO_2_ colloid. In the presence of Zn ions and tert-butanol, the crosslinking reaction was initiated, and subsequently, gels were subjected to solvent exchange and supercritical CO_2_ drying [98].

The inclusion of TiO_2_-NPs in the films modifies its physical, chemical, and biological activity, emphasizing the scope of application of these nanoparticles in food packaging by developing composites. In a study, it was observed that the addition of TiO_2_-NPs in polyethylene-based films improved the antimicrobial activity of the film [99]. Another study revealed that integrating TiO_2_-NPs on polylactic acid (PLA) substrates, cellulose nanofibers, and nanocomposites coatings reduced penetrant diffusivity while having no effect on gas barrier qualities; nonetheless, it marginally lowered the optical transparency of the film [100]. When TiO_2_-NPs (0.5, 1, and 2 wt percent) were added to potato starch films, optical transparency and tensile strength increased somewhat, whereas water vapor transmission was significantly reduced [101]. Similarly, Goudarzi et al. (2017) found that when TiO_2_-NPs at different concentrations of 1, 3, and 5% were incorporated into starch films, the hydrophobicity and thermal properties increased, whereas the tensile strength, Young’s modulus, and water vapor permeability decreased with increase in the concentration of TiO_2_-NPs [102]. TiO_2_ demonstrated ethylene scavenging activity, which lowered the rate of deterioration of fresh produce. Phothisarattana et al. (2021) showed the efficiency of TiO_2_ integrated biodegradable films in extending the shelf life of banana [103].

### 3.3. Copper Oxide (CuO) Nanoparticles for Food Packaging

CuO-NPs are the most extensively used metal oxides in food packaging, attributed to their widely effective antimicrobial properties and significant potential to inhibit the growth of bacteria, viruses, and fungi [103]. The CuO-NPs are mostly used as catalysts, polymer reinforcing agents, semiconductors, solar cells, magnetic storage media, water disinfectants, gas sensors, emission devices, and food packaging materials [104]. Many methods are employed for the fabrication of CuO-NPs, such as microwave [105], auto-combustion [106], electrochemical [107], and thermal decomposition [108]. Nanocomposites have been synthesized by adding CuO-NPs, chitosan nanofibers, and bacterial nanofibers using the chemical precipitation method [109]. In a study by Gu et al. (2018), an eco-friendly ultrasound method was used to prepare monoclinic-based CuO-NPs by utilizing extract of *Cystoseira trinodis* [49]. In another research, spherical CuO-NPs were made in situ under alkaline conditions using gelled cellulose II matrix as a template [110].

Copper has been found to be vital in the metabolism and electron transport mechanisms of living organisms. In the case of CuO-NP, the synthesis technique is critical in establishing its characteristics and finding applications in many fields, which subsequently depend upon biological activities. A study was carried out by Beigmohammadi et al. (2016) in which antimicrobial activities of packaging films, i.e., LDPE incorporated with Ag-NPs, CuO-NPs, and ZnO-NPs, against coliform in ultra-filtered cheese were determined [111]. After analysis, it was found that the coliform count decreased to 4.21 log CFU/g when stored for 4 weeks at a temperature of 4 ± 0.5 °C for each of the treatments. Another study determined the different properties of nanocomposites, such as water vapor transmission rate, UV and thermal stability, and antimicrobial properties. It was observed that the inclusion of CuO-NPs in the film improved the properties noted above along with presenting antimicrobial effects against *E. coli* and *Listeria monocytogenes* [112]. A combined antibacterial effect was obtained when CuO-NPs were incorporated in chitosan nanofibers [109]. Similar to the other cases of metal oxides, the antimicrobial activity of CuO depends upon morphology, surface area, size, structure, and oxidation states. Moreover, the packaging material properties are also affected by doping or coupling CuO-NP with other active materials such as metal or metal oxides.

Antibacterial polymeric film (APF) was developed using a variety of combinations of sodium alginate (SA) and cellulose nanowhiskers (CNW) surrounded with copper oxide nanoparticles (CuO-NPs) [113], which was used to pack fresh cut pepper (FCP). The antimicrobial activities against different strains of pathogens were tested using the disc diffusion method. From the study, it was found that the film consisting of combination CNW (0.5%)-SA (3%)-CuO-NP (5 mM) presented a significant zone of inhibition for bacteria. The zone of inhibition for *S. aureus* was at 27.49 ± 0.91 mm, *E. coli* at 12.12 ± 0.58 mm, *Salmonella* spp. at 25.21 ± 1.05 mm, *C. albicans* at 23.35 ± 0.45 mm, and *Trichoderma* spp. at 5.31 ± 1.16 mm. It was observed that when the combination was SA (1%)-CuO-NPs (1 mM), then the zone of inhibition was 21.65 ± 0.62 mm, whereas when the combination was SA (3%)-CuO- NPs (1 mM), the zone of inhibition became 12.25 ± 0.84 mm against *S. aureus*. Thus, it was concluded that with an increase in the concentration of CuO-NPs, the antimicrobial activity improved, but with the increase in the concentration of sodium alginate, diffusion of CuO-NPs in agar plates was retarded and reduced the antimicrobial action. This was attributed to the activated carbon present in the alginates, which absorbed the metal ions [114]. In this study, it was demonstrated that the presence of CuO-NP led to the antimicrobial activity of the APF; hence, the film increased the shelf life of FCP. Furthermore, the antioxidant property of the film was studied using 2,2-diphenyl-1-picrylhydrazyl (DPPH) and 3-ethylbenzothiazoline-6-sulfonic acid (ABTS) scavenging activities. It was observed that the film containing CNW (0.5%)-SA (3%)-CuO NPs (5 mM) provided the highest value for the DPPH scavenging as well as ABTS scavenging, i.e., 46.55% and 35.46%, respectively, as compared to all other films. It was due to the diffusion of CuO-NPs and CNW, wherein the CuO-NPs of the film transfer its electron density to the free radical present at the nitrogen atom in DPPH [115]. CNW contributed to the antioxidant activity by diffusion of hydrogen ions from the hydroxyl group of the glucose unit [116]. Thus, it was concluded that due to the inclusion of CuO-NP and CNW in the film, its antioxidant activity increased.

Some of the research also showed that many properties of packaging materials, including oxygen and water barrier properties, optical properties, antimicrobial properties, and bactericidal effects against Gram-positive and Gram-negative bacteria, can be modified with the hybridization of nanoparticles such as Cu/CuO-NPs, CuO-NPs/Ag-NPs, etc. A study was carried out in which the antibacterial effects of Cu-ONPs and ZnO-NPs against Gram-positive (*E. coli*) and Gram-negative (*S. aureus*) bacteria were evaluated using the Kirby Bauer Disk diffusion method, where the CuO-NPs and ZnO-NPs were developed using the wet chemical method [117]. Antibacterial activity was studied at different concentrations from 5 mg/mL to 0.01 mg/mL. It was found that CuO-NP, at a concentration lower than 1 mg/mL, did not show any antibacterial effect against *E. coli*. As the concentration increased to 1 mg/mL, it showed marginal antibacterial activity on *E. coli*. It presented good antibacterial activity at a concentration of 5 mg/mL and 2 mg/mL. Similarly, when the antibacterial activity of CuO was tested against *S. aureus*, it showed significant activity at concentrations from 5 mg/mL to 0.25 g/mL. In contrast, ZnO was not effective against *E. coli*, but it showed a significant effect against *S. aureus*. Several studies also revealed that antimicrobial properties incorporated into polymers effectively compensated for poor barrier qualities of biodegradable packaging films [118,119].

### 3.4. Silicon Dioxide (SiO_2_) Nanoparticles for Food Packaging

Silicon is one of the major solid elements found on the earth and is available in silica and silicate. According to many studies, SiO_2_–NPs, when added into different polymers, increased the mechanical strength and thermal stability [120]. It has also been observed that SiO_2_–NPs can be applied on jars, bags, and bottles as a non-sticky coating [121]. In a study by Li et al. (2016), when polypropylene (used in many packaging for proper printing) was mixed with Nano-SiO_2_ (0.09 wt%) and modified with ethylene/vinyl acetate (EVA), adsorption of ink on the polymer decreased along with an increase in the tensile strength and decrease in gas permeability [122].

SiO_2_–NPs can also be included in different coatings that are further used to prepare nanocomposites. In an experiment using the sol-gel process, a PLA-based coating was developed with the incorporation of SiO_2_–NPs using tetraethoxysilane as a precursor and 3-iso-cyanatopropyl-triethoxysilane as a coupling agent to produce a biodegradable packaging system. The biodegradable packaging film thus formed maintained good transparency and a high gas barrier property, which was 70% more than the film made from pure PLA [123]. The addition of SiO_2_–NPs in the packaging material increased corresponding antimicrobial activity. It was observed that the shelf life of shrimp packaged using LDPE-SiO_2_ packaging increased by eight days compared to other samples stored otherwise [124]. A decreased oxygen permeability reduced oxygen levels in the package headspace that accelerated quality deterioration in packaged shrimp. Improving barrier properties would increase the utilization of biodegradable packaging to preserve quality of packaged products [125].

## 4. Conclusions

A brief overview of the properties of food packaging films using various metal nanoparticles is included in this study. The different studies revealed that inorganic nanoparticles have been extensively investigated along with efficient demonstrations of antimicrobial activities. Antimicrobial emulsions based on nanomaterials would be useful for decontaminating food packaging or food products. The antimicrobial activities of nanoparticles incorporated packaging film are primarily due to their high surface area to volume ratio. Nanotechnology based sensors can also be implemented to detect and identify food contamination. Gold, copper, and silver have been the most exploited elements for achieving excellent packaging systems causing shelf-life extension of the food material. According to some investigations, incorporating nanoparticles increased the oxygen barrier properties and reduced the water vapor transmission rate of conventional flexible food packaging. Therefore, improved plastic film and coatings infused with nanoparticles for food packaging and storage would allow for more widespread and efficient transportation and storage of food products. However, with evolving technologies, the mechanisms of production and application can be further improved, thus easing the commercialization of the techniques. Other metals can also be exploited, making the process and technology more viable and accessible. Hence, there is still a lot of untapped potential in nanotechnology, which should be further explored and employed in food engineering and packaging technology.

## Figures and Tables

**Table 1:** Summary of various studies on the application of coatings with silver nanoparticles to store various samples under different conditions and the corresponding tested strains for assessment of antimicrobial activity.

Coating	Samples	Storage Conditions	Tested Strains	Result	References
Alginate coating with AgNPs	Shiitake mushrooms	4 ± 1 °C	Mesophilic, psychrophilic, Pseudomonas, yeast, and molds	Extended shelf life to 16 days	[32]
Chitosan impregnated with AgNPs	Strawberries	7 °C for 25 days	*Botrytis cinerea*	10% fungal decay (coated samples) 90% (uncoated samples)	[33]
Silver montmorillonite nanoparticles and sodium alginate coating	Fresh cut carrots		-	Prolonged shelf life of 70 days (coated samples) as compared to 4 days (uncoated samples)	[34]
Gum Arabic based coating dispersed with AgNPs	Green bell peppers	21 days at 7 °C and 20 °C	Aerobic bacteria	Coated samples inhibited the growth of aerobic bacteria with improved appearance and delayed microbial decay compared to uncoated ones; also remains marketable even after 21 days of storage	[35]
Carboxymethylcellulose and guar gum-based coating with AgNPs	Kinnow mandarin	10 °C and 4 °C	Psychrotrophic aerobic yeast and mold	Both coatings limit the growth of yeast and mold at 10 °C but completely prevent at 4 °C; also shelf life increased to 60 days (10 °C) and 120 days (4 °C)	[36]
Agar coating enriched with AgNPs	Citrus aurantifolia			Extended shelf life up to 9 days	[37]
Hydrosol based on chitosan, hydroxypropyl methylcellulose, and nanosilver	Meat surface	4 °C for 4 weeks	Undesirable bacteria	The reduction of bacteria was 2.5 log CFU/g compared to the uncoated samples	[38]
Active pullulan edible packaging with silver nanoparticles	Vacuum-packaged ready-to-eat turkey deli meat	Stored at refrigerated temperature	Food-borne pathogens *Listeria monocytogenes* and *Staphylococcus aureus*	Reduction of count from 7 log CFU/g to below the detection limit during 14 days of storage at 4 °C	[39]
Edible coating containing silver nanoparticles	Vacuum-packaged sausages	10 °C	*lactic acid bacteria*	Can inhibit bacterial activity till 30 days, thus increasing shelf life	[40]
Agar hydrosol with nanosilver	Cheese	10 °C.	*Pseudomonas* spp. And *coliforms bacteria*	Inhibited growth of spoilage bacteria and also increased the shelf life to 6 days compared to the untreated one (1.5 days)	[41]
Silver nanoparticles stabilized with glutathione	--	Vacuum sealing and Modified atmospheric packaging	Multidrug-resistant strains of *Campylobacter*	Susceptibility of Campylobacter strains to silver nanoparticles at a concentration of less than or equal to 9.85 micrograms/mL	[42]
Composite films of gelatin, chitosan, polyethylene, and silver nanoparticles	Red Grapes	--	Molds	Extended the storage life by 14 days	[43]
Coating of silver nanoparticles stabilized with cellulose nanocrystals on paper	Strawberries	--	*E. coli* and *S. aureus*	Augmented the shelf life up to 7 days	[44]
Green organic-inorganic hybrid nanofibers by the conglomeration of poly(vinyl alcohol) and silver nanoparticles	Lemon and strawberries	Room temperature	*B. subtilis*, *S. aureus*, *E. coli* and *P. aeruginosa*	Repressed the proliferation of pathogens up to 10 days	[45]
Biobased and compostable composite film of PVA-montmorillonite K10 clay ginger extract mediated AgNPs	Chicken sausages	4 °C	*S. Typhimurium* and *S. aureus*	Depreciation in the growth of pathogens when packed in the pouches made from composite films	[46]
AgNPs incorporated in agar and banana-based films			*E. coli* and *L. monocytogenes*	Superior bacteriocidal property	[47]
Amazonian tuber starch-based films with AgNPs	Camu Camu fruit		*S. aureus* and *E. coli*	Good inhibition towards pathogens; in addition, ripening was delayed	[48]

**Table 2:** Different materials with gold nanoparticles and their effects on different microorganisms.

Coating/Packaging	Food Samples	Experimental Condition	Antimicrobial Activity	Result	References
Incorporation of AuNPs and grapheme oxide separately on PVA (Polyvinyl alcohol) composite films	Banana	Room temperature for 5 days	*E. coli*	Zone of inhibition for *E. coli* increased to 10 mm for PVA-Glyoxal-AuNPs as compared to 0 mm for PVA alone	[57]
Blend of 3-aminopropyltrimethoxysilane, chitosan, and gold nanoparticles	--	--	*S. Typhimurium*	Exhibited excellent activity against Salmonella sp. owing to the interaction of the constituents with cell membrane leading to their death	[58]
AuNPs dispersed on zeolites	--	--	*E. coli* and *S. typhi*	Materials contained particles sized 5 nm on the surface eliminated 95% of both microorganisms	[51]
Combination of bacteriocin and gold nanoparticles	--	--	*Micrococcus luteus*, *Bacillus cereus*, *S. aureus*, *E. coli*	Antibacterial activity increased against food-spoiling bacteria	[53]
Gold nanoparticles with nisin	--	--	*E. coli*, *S. aureus*, *B. cereus*, *K. pneumonia*, *Proteus mirabilis*	Antibacterial activity against food-spoilage microorganisms	[59]
AuNPs based colorimetric sensor	Meat and fish spoilage	Dimethyl sulfide and histamine are two significant volatile biogenic markers	--	Able to detect histamine at 0.035 ppm and dimethyl sulfide at 0.5 ppm	[60]

**Table 3:** Methods of fabrication of ZnO nanoparticles.

Shapes	Methods	References
Flower	Solution process at low temperature (90 °C) using zinc acetate dehydrate and NaOH	[87]
Flower, prism, snowflakes	Solution process at high temperature (180 °C for 13 h)	[88]
Prism like and prickly sphere like	Decomposition method at 100 °C for 13 h	[83]
Spherical	Non hydrolytic solution process using zinc acetate	[89]
Spherical	Soft chemical solution process	[90]
Nanorods of hexagonal prismatic and hexagonal pyramid like	Hydrothermal treatment with stabilizing agents	[75]
Nanowires	UV light decomposition process	[91]

## Data Availability

Not applicable.
